# Distribution of Ammonia-Oxidizing Archaea and Bacteria in the Surface Sediments of Matsushima Bay in Relation to Environmental Variables

**DOI:** 10.1264/jsme2.ME11218

**Published:** 2011-12-27

**Authors:** Tomoko Sakami

**Affiliations:** 1 Tohoku National Fisheries Research Institute, Fisheries Research Agency, 3–27–5, Shinhama-cho, Shiogama, Miyagi 985–0001, Japan

**Keywords:** ammonia-oxidizing archaea, ammonia-oxidizing bacteria, estuarine sediment, organic matter degradation, total phosphorus

## Abstract

Ammonia oxidization is the first and a rate-limiting step of nitrification, which is often a critical process in nitrogen removal from estuarine and coastal environments. To clarify the correlation of environmental conditions with the distribution of ammonia oxidizers in organic matter-rich coastal sediments, ammonia-oxidizing archaea (AOA) and bacteria (AOB) ammonia monooxygenase alpha subunit gene (*amoA*) abundance was determined in sediments of Matsushima Bay located in northeast Japan. The AOA and AOB *amoA* copy numbers ranged from 1.1×10^6^ to 1.7×10^7^ and from 7.1×10^5^ to 7.6×10^6^ copies g^−1^ sediment, respectively. AOA and AOB *amoA* abundance was negatively correlated with dissolved oxygen levels in the bottom water. AOA *amoA* abundance was also correlated with total phosphorus levels in the sediments. On the other hand, no significant relationship was observed between the *amoA* abundance and ammonium, organic matter (ignition loss), or acid volatile sulfide-sulfur levels in the sediments. These results show the heterogeneous distribution of ammonia oxidizers by the difference in environmental conditions within the bay. Moreover, AOA *amoA* diversity was relatively low in the area of high AOA *amoA* abundance, suggesting the variability of AOA community composition.

Ammonia oxidization is a rate-limiting step in nitrification; furthermore, it is related to other processes such as denitrification and anaerobic ammonium oxidation that are important for nitrogen removal in estuarine ecosystems (7, 9). The past decade has seen an increasing number of studies on ammonia-oxidizing microbial abundance and diversity via the detection of the ammonia monooxygenase alpha subunit gene (*amoA*) in various environments (7, 9, 27). In addition to ammonia-oxidizing bacteria (AOB), which have previously been considered to be solely responsible for the ammonia-oxidizing step, ammonia-oxidizing archaea (AOA) have been discovered (15), and are important in ammonia oxidation in many natural habitats (7, 27).

Compared with AOB, AOA appear to be more important in the nitrogen cycle of marine ecosystems (7) because AOA *amoA* copy numbers are 10–100 times greater in coastal environments than AOB *amoA* copy numbers (26). AOA in estuarine environments are also associated with nitrification (4). AOA appear to be better adapted to life in oligotrophic environments than AOB because the only isolated AOA strain, “*Nitrosopumilus maritimus*” SCM1, could grow in much lower ammonia levels than those required for the growth of cultured AOB (3); the strain also had marked and highly specific substrate affinity (17); however, the factors that determine the distribution and contribution of both types of ammonia oxidizers have not been sufficiently clarified in organic matter-rich sediments (7). AOA are also found in coastal sediments with high ammonia levels (2, 4, 6, 9, 16, 22), but some reports have indicated that AOB are the major contributors to nitrification in estuarine sediments (16, 18). In addition to substrate concentrations, AOA are considered to tolerate lower dissolved oxygen (DO) levels than AOB (7). DO levels at the bottom of water bodies is often low during summer and fall in eutrophic coastal environments; therefore, DO levels are also believed to contribute to the distribution of ammonia oxidizers.

The purpose of this study was to clarify the correlation of environmental conditions with the distribution of ammonia oxidizers in bottom sediments in an organic matter-rich bay. We examined AOB and AOA *amoA* abundance in Matsushima Bay, Japan, and determined its correlation with chemical and microbial parameters.

## Materials and Methods

### Sample collection and chemical analysis

**Fig. 1:**
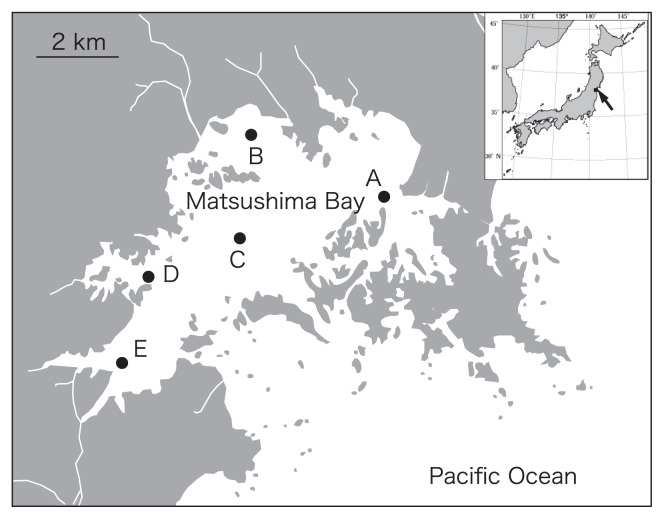
Map showing the location of the sampling sites in Matsushima Bay.

Matsushima Bay is a small, shallow, enclosed bay located on the Pacific coast of northeast Japan (Fig. 1). Organic matter accumulates on bottom sediments and DO levels in these areas often decrease during summer and fall. The sediments are mainly composed of silt. Sediment was sampled at five sites in Matsushima Bay in October 2009. Water temperature and salinity were measured at depths of 50 cm below the surface and 20 cm above the bottom using a portable device (ACT20-D; Alec, Kobe, Japan). Two cores were taken at each site. The overlying water from the sampled core was siphoned out and fixed to measure DO levels by the Winkler method (24). The surface sediment (top 1 cm) was stored in a sterile plastic tube and transported to the laboratory under cool and dark conditions within 4 h after collection. Subsamples for DNA extraction were frozen immediately at −70°C.

Acid volatile sulfide-sulfur (AVS-S) levels were determined using an H_2_S-absorbent column (Gastech, Kanagawa, Japan) after extraction from the sediment samples in 5 N H_2_SO_4_ according to the manufacturer’s instructions. Ignition loss (IL) was determined by igniting the sediment samples at 550°C for 4 h. Total phosphorus (TP) levels were determined by extracting orthophosphate from the ignited sediment samples by boiling for 15 min in 1 N HCl and colorimetric measurement of the levels (1). Ammonium levels were determined colorimetrically after extraction from the sediment samples in 1 M KCl (13).

### Microbial parameters

The abundance of ammonia oxidizers was measured by the most probable number (MPN) method using media containing 0.23 mM ammonium sulfate as the substrate (14). β-D-glucosidase activity in the sediment samples was determined as a parameter of microbial organic matter degradation using a fluorophore-labeled analog substrate 4-methylumbelliferyl-β-D-glucoside (MUF-β-glucoside) (12). This analog substrate was added to duplicate sediment samples suspended in phosphate buffer (pH 8.0) at a final concentration of 100 μM and incubated for 20–60 min at 37°C. Periodically, subsamples were taken and centrifuged in bicarbonate buffer (pH 10). Increases in the fluorescence of the supernatants were measured using a spectrophotometer (RF-5300PC; Shimadzu, Kyoto, Japan) and standard MUF solutions (21).

### DNA Extraction

DNA was extracted from 0.4–0.6 g of the sediment sample using the Fast DNA SPIN Kit for soil (MP Biomedicals, Solon, OH, USA) according to the manufacturer’s instructions. The extracted DNA was dissolved in 50 μL TE buffer and stored at −30°C until further analysis.

### Quantitative PCR

To determine *amoA* gene copy numbers, quantitative PCR (qPCR) was performed using the ABI StepOne™ System (Applied Biosystems/Life Technologies, Carlsbad, CA, USA) with SYBR *Premix Ex Taq* (Takara Bio, Otsu, Japan) and the primers crenAMO F/crenAMO R for crenarchaeal *amoA*(15) and amoA-1F/amoA-2R for betaproteobacterial *amoA*(19). Each PCR reaction mixture (10 μL) contained 1 μL template DNA solution, 0.2 μg μL^−1^ bovine serum albumin, 0.2 μM of each primer, and 5 μL SYBR *Premix Ex Taq*. PCR was performed with initial denaturation at 95°C for 30 s, followed by 40 cycles at 95°C for 15 s, 57°C for 15 s, and 72°C for 30 s, followed by melting curve analysis (2). Fluorescence intensity was measured at 72°C. Standards consisted of cloned AOA or AOB *amoA* fragments (from biofilter materials) that contained the region of each primer set. Melting curve analysis ensured that only products of the desired melting temperature were generated.

### Cloning of archaeal *amoA*

The primers crenAMO F/crenAMO R were used to amplify archaeal *amoA*s from the sediment DNAs. PCR was performed using *Ex Taq* HS polymerase (Takara Bio) with initial denaturation at 94°C for 2 min, followed by 30 cycles of denaturation at 94°C for 45 s, annealing at 58°C for 30 s, and extension at 72°C for 45 s; final elongation was performed at 72°C for 7 min. The amplified archaeal *amoA*s were cloned using the TA cloning kit (DynaExpress DNA Ligation Kit ver. 2; Biodynamics Laboratory, Tokyo, Japan). The M13F/M13R PCR products were sequenced by a cycle sequencing reaction using a sequencing kit (BigDye Terminator Cycle version 3.1; Applied Biosystems/Life Technologies) on a capillary DNA sequencer (ABI 3100; Applied Biosystems/Life Technologies).

Operational taxonomic units (OTUs) were defined as groups of nucleotide sequences that differed by 2% or less. The Shannon–Wiener index (*H′*) was used as the diversity index (23). The determined archaeal *amoA* sequences were aligned using the GENETYX-MAC Ver.13 software package (Genetyx, Tokyo, Japan) and compared with those available from the DDBJ/EMBL/GenBank databases using nucleotide-nucleotide BLAST software. Alignment editing and phylogenetic analyses (by the neighbor-joining method) were implemented using MEGA version 4.1 software (25).

### Nucleotide sequence accession numbers

The *amoA* sequences were submitted to the DDBJ/EMBL/GenBank and have been assigned accession numbers AB629995 to AB630019.

## Results

### Environmental parameters

The water temperature and salinity changed gradually from the end (Site A) to the mouth (Site E) of the bay, although the difference was minimal (Table 1). The lowest salinity in the bottom water was 29.8 and observed at Site A, which was away from the river mouth, suggesting that the effect of river water discharge was small at the examined sites. The DO level in the bottom water was 97 and 100% of saturation levels at sites A and C, respectively, whereas bottom DO levels decreased at sites B, D, and E, which had 74%, 80%, and 81% of saturation levels, respectively. Furthermore, DO levels in the surface water were as low as those in the bottom water at site E. The mean ammonium levels in the sediment samples at sites B, D, and E were 2–5 times higher than those in sediment samples at sites A and C (Table 2). Moreover, the mean β-D-glucosidase activity was 2–3 times higher at sites B and E than at the other sites. Ammonium levels and β-D-glucosidase activity negatively correlated with bottom DO levels (Pearson’s correlation: *r*=−0.79 and *r*=−0.73 for the ammonium content and β-D-glucosidase activity, respectively; *p*<0.05). Differences in TP levels among the sediment samples were relatively small compared to those in ammonia levels, but TP levels were higher at sites B and D (60–75 μmol g^−1^) than at the other sites (43–55 μmol g^−1^). The IL values were >14% in all samples and did not show any clear tendencies. AVS-S levels in the sediment samples were low at site A (0.17 mg S g^−1^ on average) and high at site C (0.66 mg S g^−1^ on average).

### Correlation coefficients of environmental parameters with abundance of ammonia oxidizers

**Fig. 2:**
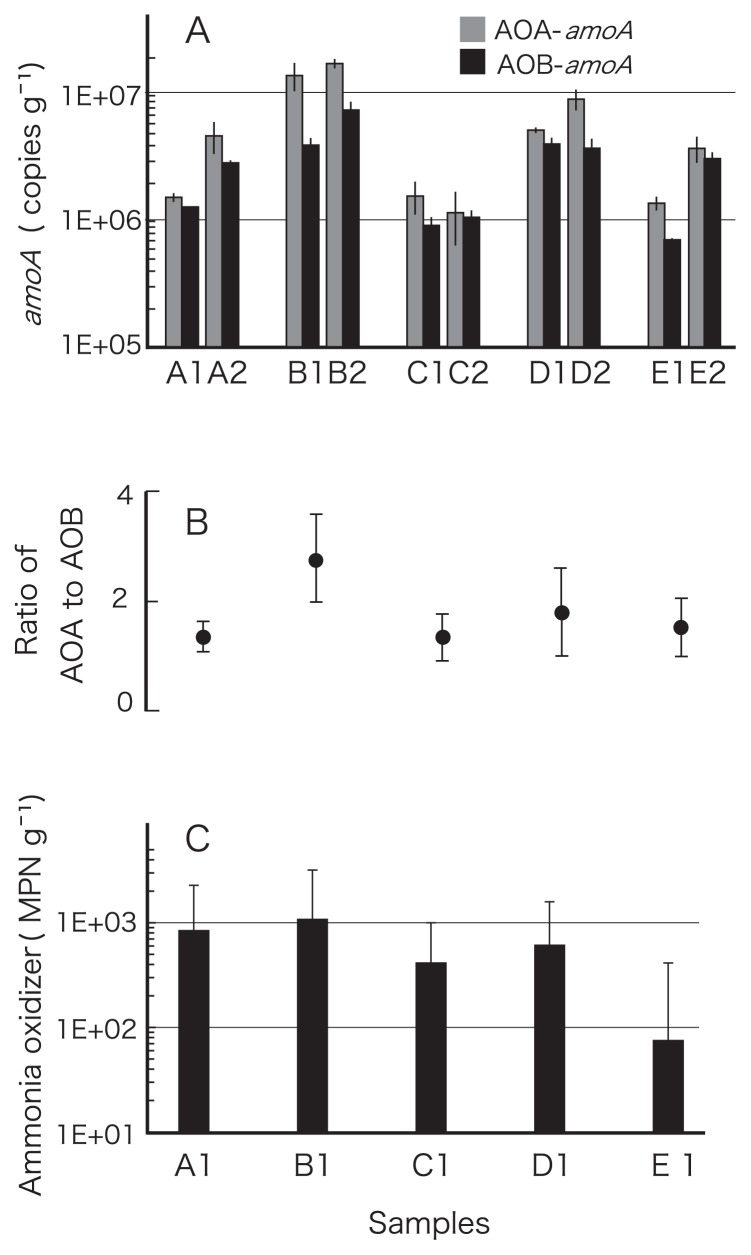
Abundance of ammonia-oxidizers in the surface sediments of Matsushima Bay. (A) The mean of archaeal and bacterial *amoA* copy numbers. Error bars represent standard deviations of triplicate qPCR determinations. (B) The mean of relative archaeal and bacterial *amoA* abundance at each sampling site. (C) MPN of ammonia-oxidizers. Error bars represent 95% confidence limits.

The AOA and AOB *amoA* copy numbers ranged from 1.1×10^6^ to 1.7×10^7^ and from 7.1×10^5^ to 7.6×10^6^ copies g^−1^ sediment, respectively. The mean AOA abundance at site B was greater than at the other sites (Student’s t-test; *p*<0.05) (Fig. 2A). The mean AOB abundance appeared to be greater at site B but this was not significant. The ratio of AOA to AOB *amoA* abundance ranged from 1.0 to 3.4 and was high at site B (Fig. 2B). MPN of ammonia oxidizers was low at sites C and E, although the difference was not significant (Fig. 2C).

AOA and AOB *amoA* abundance negatively correlated with DO levels in the bottom water (Table 3). AOA *amoA* abundance also correlated with TP levels in the sediment samples. Other environmental parameters did not show significant correlations with gene abundance.

### Diversity of AOA *amoA*

**Fig. 3:**
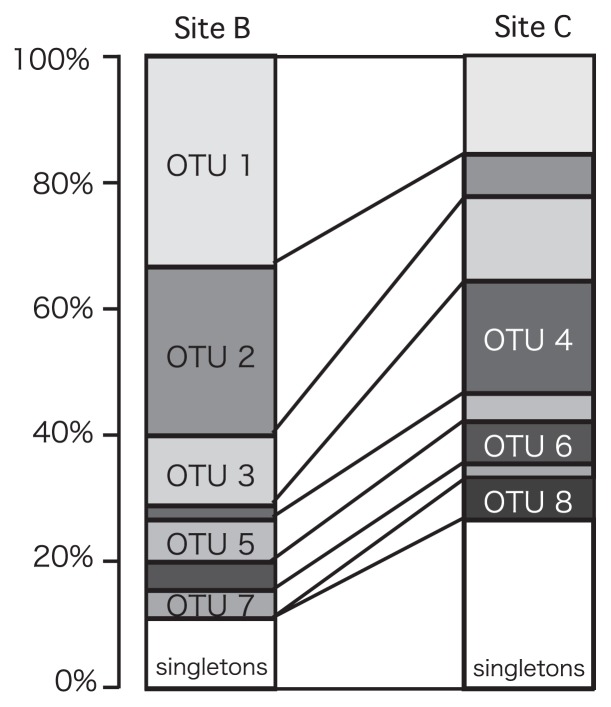
Composition of OTUs of archaeal *amoA* clones recovered from surface sediment samples in Matsushima Bay.

**Fig. 4:**
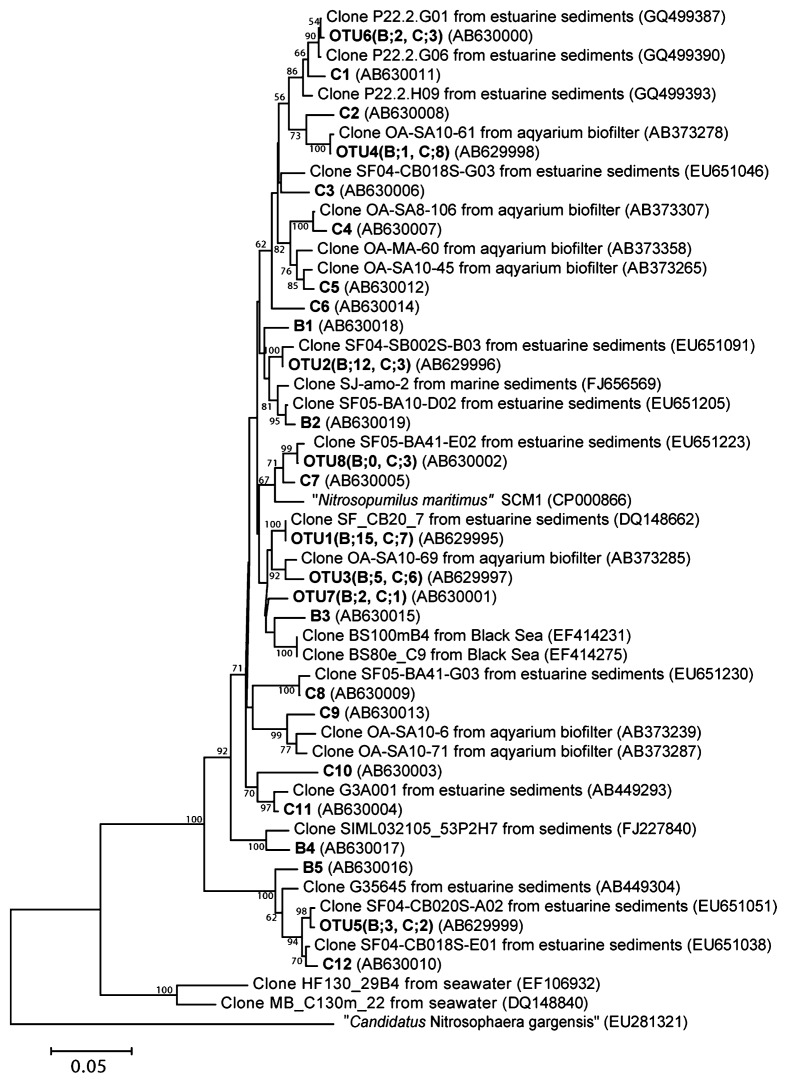
Neighbor-joining phylogenetic tree of archaeal *amoA* gene sequences (456 bp). Bold indicates the representative *amoA* clones obtained in this study. Letters and numbers in parentheses appended to each OTU denote clone numbers detected from each site. B1–B5 and C1–C12 denote singleton clones from sites B and C. Scale bar represents an estimated sequence divergence of 5%. Numbers at nodes indicate bootstrap values (>50%).

Twelve OTUs were recovered at site B and 20 OTUs at site C from 46 sequenced AOA *amoA* clones (Fig. 3). H′ at site B (H′=2.5) was smaller than that at site C (H′=3.3). All AOA *amoA* clones fell phylogenetically into the “water column/sediment” cluster originally defined by Francis *et al.*(8), which contains the ammonia-oxidizing archaeon “*N. maritimus*” (Fig. 4). The proportions of OTU 1 and OTU 2 were greater at site B than those at site C. These OTUs were most closely related (with 99% similarity) to a sequence from estuarine sediments.

## Discussion

Caffrey *et al.*(4) examined the nitrification activity and abundance of ammonia oxidizers in various estuarine sediments. They suggested that nitrification may be maximized when DO is present and labile organic matter is high but pore water sulfide levels are low. In Matsushima Bay, AOA and AOB *amoA* abundance negatively correlated with DO levels in the bottom water. Ammonia oxidization is certainly an aerobic process; however, it has been shown that AOA or some specific ecotypes tolerate a wide range of oxygen levels (8). AOA *amoA* is often detected in environments with low DO levels, as low as <1 μM in one case (5), and AOA *amoA* abundance did not differ at DO levels ranging from 0.1–0.2 mM in aquifer sediments (22). Moreover, Hatzenpichler *et al.*(10) reported that cultured AOA (“*Candidatus* Nitrososphaera gargensis”) showed ammonia-oxidizing and stoichiometric nitrite-producing activities at a DO level of 0.2 mM. DO levels in the pore water should be lower than in the bottom water, which was approximately 180 μM; nevertheless, AOA were not considerably affected by low DO levels found in the examined areas. On the other hand, AOB are generally more sensitive to decreases in DO levels (7). In this study, AOB *amoA* abundance did not decrease at sites B and D where bottom DO levels were low, indicating that decreases in DO levels did not have a considerable effect on the AOB community. In estuarine sediments, it has been shown that sulfide levels rather than DO levels are more likely to affect ammonia-oxidizing activity, AOA and AOB abundance, and community composition (4, 7); however, no significant correlation was observed between AVS-S levels and *amoA* abundance in this study, suggesting that the sulfide level was a negative factor for AOA and AOB abundance in the examined area.

Ammonium levels and β-D-glucosidase activity in the sediment samples negatively correlated with bottom DO levels, indicating that organic matter was actively degraded and ammonia was generated where the bottom DO level was low. Ammonium supply is likely to be an important factor in the distribution of ammonia oxidizers in the bay, although a direct significant correlation was not observed between *amoA* gene abundance and ammonia levels. Generally, AOA are predominant in oligotrophic oceanic environments. Martens-Habbena *et al.*(17) showed that the only isolated AOA strain “*N. maritimus*” SCM1, had marked and highly specific affinity for reduced nitrogen, enabling it to successfully compete with heterotrophic bacterioplankton and phytoplankton in oceanic seawater. The half-saturation constant for ammonium uptake (*K*_m_) was 0.133 μM of total ammonia, which is 2–4 orders of magnitude smaller than that of cultured AOB (17). In this study, the ammonia level of pore water calculated using the water content in the sediment ranged from 0.4–2.4 mM. Although the ammonia level of *in situ* pore water may not be exactly the same as the calculated level, it is similar to the *K*_m_ of AOB (17). This suggests that ammonia levels are sufficiently high for AOB to compete with AOA, and therefore, the ratio of AOA to AOB was low compared to in seawater and soils (7). Such a low ratio has also been reported in other eutrophic estuarine sediments (4). The cultured AOA strain SCM1 grew well in media containing 0.5 mM ammonium (15), but its ammonium uptake activity was inhibited at high ammonium levels (>2 mM) (17). In this study, the calculated ammonia levels in the pore water were 1.2 and 2.4 mM at site B where AOA *amoA* abundance was high, indicating that AOA could still grow in such high ammonia levels. Some AOA ecotypes may adapt to the high ammonia levels in eutrophic estuarine sediments (7).

In addition to ammonium levels, AOA *amoA* abundance also correlated with TP levels in the sediments. Particularly at site E, DO levels, β-D-glucosidase activity, and ammonium levels were similar to or greater than those at sites B and D, but TP levels were as low as at sites A and C, suggesting that the low phosphorus content may be related to the low *amoA* abundance in these sediments. A positive correlation has also been reported between crenarchaeal 16S rRNA gene copies, most of which are ammonia oxidizers, and phosphate levels in coastal seawater, although inorganic nitrogen levels were also correlated in the reported case (20).

AOA *amoA* diversity was lower at site B, where gene abundance was high, compared to site C, where gene abundance was low. OTU 1 and OTU 2 predominated at site B, indicating that these phylotypes may be present in increased numbers in this area. Previous studies have reported that AOA *amoA* diversity is affected by net primary production or nitrogen and ammonium levels in the sediment as well as by temperature and salinity (6, 20).

In conclusion, AOA and AOB *amoA* abundance negatively correlated with DO levels in the bottom water in Matsushima Bay. Ammonia supply by organic matter degradation appeared to be related to AOA and AOB distribution. In addition, AOA *amoA* abundance positively correlated with TP levels in the sediments, suggesting that the phosphorus supply may be involved in sedimentary ammonia-oxidization in this bay. AOA *amoA* diversity was relatively low in areas of high AOA *amoA* abundance, suggesting that certain *amoA* phylotypes may increase in low DO and high ammonia environments. Further studies should be conducted on the environmental factors associated with AOA and AOB distributions to clarify the dynamics of the ammonia oxidization process in organic matter-rich estuarine sediments.

## Figures and Tables

**Table 1:** Physical properties of the surface and bottom water at the sampling sites

Site	Depth (m)		Temp. (°C)	Salinity	DO (μM)
A	4.5	Surface	17.2	27.6	241
		Bottom	18.6	29.8	237
B	2.5	Surface	16.6	28.0	241
		Bottom	18.6	30.9	179
C	4.5	Surface	16.9	28.8	246
		Bottom	18.8	32.1	241
D	4.5	Surface	17.3	30.0	232
		Bottom	18.7	31.8	192
E	7.2	Surface	18.1	30.8	201
		Bottom	19.0	32.7	192

Abbreviations: DO, dissolved oxygen

**Table 2:** Chemical and microbial parameters of the sediment samples

Site	Sample #	NH_4_^+^ (μmol g^−1^)	TP (μmol g^−1^)	IL (%)	AVS-S (mg S g^−1^)	β-D-glucosidase activity (μmol h^−1^ g^−1^)
A	A1	1.7	44	14	0.23	0.39
	A2	1.8	55	14	0.10	0.46
B	B1	4.0	70	15	0.27	1.2
	B2	9.4	60	16	0.60	0.98
C	C1	1.0	49	16	0.63	0.38
	C2	1.8	43	14	0.68	0.31
D	D1	3.3	60	15	0.70	0.44
	D2	4.0	75	15	0.49	0.37
E	E1	6.9	48	15	0.46	1.1
	E2	7.1	43	14	0.48	0.94

Abbreviations: TP, total phosphorus levels; IL, ignition loss; AVS-S, acid volatile sulfide-sulfur levels

**Table 3:** Pearson’s correlation coefficients of the environmental parameters with *amoA* abundance and their ratios in sediment samples

	AOA	AOB
Bottom DO	−0.68*	−0.66*
NH_4_^+^	0.55	0.61
TP	0.73*	0.60
IL	0.33	0.27
AVS-S	−0.03	0.10
β-D-glucosidase activity	0.51	0.35

* *p*<0.05 (*n*=10)

Abbreviations: TP, total phosphorus levels; IL, ignition loss; AVS-S, acid volatile sulfide-sulfur levels; DO, dissolved oxygen
